# Introduction of formula feeding induces subclinical inflammation and altered chromatin structure in the intestine of preterm pigs

**DOI:** 10.1186/2194-7791-2-S1-A6

**Published:** 2015-07-01

**Authors:** Rhea Willems, Verena Rybicki, Pingping Jiang, Per Torp Sangild, René Liang Shen, Kai O Hensel, Stefan Wirth, Jan Postberg, Andreas C Jenke

**Affiliations:** Department of Neonatology, HELIOS Children´s Hospital, Centre for Biomedical Education and Research (ZBAF), Witten/Herdecke University, Wuppertal, Germany; Comparative Pediatrics and Nutrition, Dept. Clinical Veterinary and Animal Science, and Copenhagen University Hospital (Rigshospitalet), University of Copenhagen, Copenhagen, Denmark

## Aim

Whereas enteral nutrition has been recognized to play an important role for the development of necrotizing enterocolitis (NEC), the exact pathomechanism is still under debate [1, 2]. In this study we aimed to analyze how introduction of enteral foods affects intestinal gene regulation and chromatin structure of different inflammatory and pattern recognition genes (i.e. IL8, TLR2, TLR4, REG3A) in premature pigs before any clinical symptoms of NEC.

## Methods

In total 15 preterm piglets were either provided with total parenteral nutrition (n = 5) or minimal enteral nutrition - bovine colostrum (n = 5) or preterm infant formula (n = 5). Gene expression analyses of the genes mentioned above were performed by quantitative PCR. Changes in chromatin conformation were assessed by endonuclease hypersensitivity assays. Functional studies analyzing the influence of chromatin conformation changes on the inflammatory response upon stimulation by lipopolysaccharide (LPS) were performed in CaCo-2 cells using the histone deacetylase inhibitor Trichostatin A (TSA).

## Results

Enteral feeding induced a significant up-regulation of pro inflammatory genes such as IL8 and TLR 4 in the absence of any clinical symptoms of NEC. Those effects were more distinct in the formula fed subgroup compared to piglets who received colostrum. Most up-regulated genes, particularly IL8 and TLR4, were associated with endonuclease hypersensitive regions and were thus located in de-condensed, active chromatin. In consistence with this finding, TSA pretreated Caco-2 cells exhibit a significant higher IL 8 up-regulation after LPS exposure compared to controls.

## Conclusion

Enteral feeding in general and formula in particular influences the regulation of key inflammatory genes and their respective epigenetic signatures in the premature gut. This might contribute to the increased susceptibility to NEC in premature infants. In consequence, more studies are urgently required to optimize enteral nutrition in preterm infants.
